# Ethylene Induces a Rapid Degradation of Petal Anthocyanins in Cut *Vanda* ‘Sansai Blue’ Orchid Flowers

**DOI:** 10.3389/fpls.2019.01004

**Published:** 2019-08-09

**Authors:** Sudarat Khunmuang, Sirichai Kanlayanarat, Chalermchai Wongs-Aree, Shimon Meir, Sonia Philosoph-Hadas, Michal Oren-Shamir, Rinat Ovadia, Mantana Buanong

**Affiliations:** ^1^Division of Postharvest Technology, School of Bioresources and Technology, King Mongkut’s University of Technology Thonburi (Bangkhuntien) Thakam, Bangkok, Thailand; ^2^Postharvest Technology Innovation Center, Office of the Higher Education Commission, Bangkok, Thailand; ^3^Department of Postharvest Science, Agricultural Research Organization (ARO), The Volcani Center, Rishon LeZion, Israel; ^4^Department of Ornamental Plants and Agricultural Biotechnology, Agricultural Research Organization (ARO), The Volcani Center, Rishon LeZion, Israel

**Keywords:** anthocyanidins, color fading, cyaniding, delphinidin, ethylene sensitivity, peroxidase activity, senescence symptoms, cut *Vanda* orchid flowers

## Abstract

Ethylene plays a major role in the regulation of flower senescence, including in the ethylene-sensitive *Vanda* ‘Sansai Blue’ orchid flowers. This cut flower is popular in Thailand due to its light blue big size florets possessing a beautiful shape pattern. In the present study, we further examined the rapid ethylene-induced process of active anthocyanin degradation in cut *Vanda* ‘Sansai Blue’ flowers, which occurred much before detection of other typical senescence-related symptoms. For this purpose, the cut inflorescences were exposed to air (control), 1 or 10 μl L^−1^ ethylene for 24 h, or to 0.2 μl L^−1^ 1-methylcyclopropene (1-MCP) for 6 h followed by 10 μl L^−1^ ethylene for 24 h at 21°C, and the effects of these treatments on various parameters were assayed. While the fading-induced effect of ethylene was not concentration-dependent in this range, the ethylene treatment significantly reduced the flower vase life in a concentration-dependent manner, further confirming the separation of the bleaching process from senescence. Exposure of the inflorescences to 1-MCP pre-treatment followed by 10 μl L^−1^ ethylene, recovered both inflorescence color and anthocyanin content to control levels. Quantification of total anthocyanin content, performed by HPLC analysis on the basis of cyanidin-3-glocuside equivalents, showed that ethylene reduced and 1-MCP recovered the anthocyanins profile in non-hydrolyzed anthocyanin samples of *Vanda* ‘Sansai Blue’ florets, assayed at half bloom and bloom developmental stages. The results showed that the ethylene-induced color fading, observed immediately after treatment, resulted from a significant reduction in the levels of the two main anthocyanidins, cyanidin and delphinidin, as well as of other anthocyanidins present in low abundance, but not from changes in the levels of flavonols, such as kaempferol. This anthocyanin degradation process seems to operate *via* ethylene-increased peroxidase activity, detected at the bud stage. Taken together, our results suggest that the ethylene-induced rapid color bleaching in petals of cut *Vanda* ‘Sansai Blue’ flowers is an outcome of *in-planta* anthocyanin degradation, partially mediated by increased peroxidase activity, and proceeds independently of the flower senescence process.

## Introduction

The plant hormone ethylene (Bleecker and Kende, 2000) plays a vital role in the regulation of flower senescence, manifested in a range of symptoms including wilting, discoloration, bud degeneration, and abscission (Reid and Wu, 1992), which also occur in senescing orchid flower species (Goh et al., 1985; Woltering and Van Doorn, 1988). The responses to ethylene vary widely between species (Reid and Wu, 1992), although they are often consistent within families or subfamilies (Van Doorn, 2001). The *Orchidaceae* is classified as one of the ethylene-sensitive flower families, with a variable sensitivity to ethylene among the species and cultivars (Akamine, 1963; Burg and Dijkman, 1967; Goh et al., 1985; Woltering and Van Doorn, 1988). *Cattleya, Paphiopedilum*, *Dendrobium*, *Phalaenopsis*, and *Cymbidium* orchids were found to be highly sensitive to ethylene, which caused color fading and wilting of sepal tips, as well as bud and flower abscission (Woltering and Van Doorn, 1988; Porat et al., 1995; Ketsa and Rugkong, 2000). Additionally, *Cymbidium* orchids showed a dramatic response to exogenous ethylene, including induction of anthocyanin formation in female reproductive parts (Goh et al., 1985). Thus, the variation in postharvest life can partly be ascribed to differences in endogenous ethylene biosynthesis, as well as to differences in sensitivity to endogenous and exogenous ethylene (Goh et al., 1985; Woltering and Van Doorn, 1988).

1-Methylcyclopropene (1-MCP), an effective blocker of ethylene perception, is considered to bind to the ethylene receptor irreversibly, resulting in the inhibition of ethylene action (Serek et al., 1994; Sisler et al., 1996). 1-MCP prevents damage from exogenous ethylene in numerous potted plants and cut flower species (Serek et al., 1994, 2006; Serek and Sisler, 2001). Application of 1-MCP suppressed 1-aminocyclopropane-1-carboxylic acid oxidase (ACO) activity and ethylene production in *Cattleya alliance* orchids (Yamane et al., 2004). 1-MCP also delayed senescence in *Cymbidium* flowers with damaged pollinia, thereby extending their vase life, protected the flowers from the deleterious effects of exogenous ethylene, and prevented premature flower senescence generated by the damaged pollinia (Heyes and Johnston, 1998).

The *Vanda* ‘Sansai Blue’ orchid is a hybrid of *V. Crimson Glory* × *V. coerulea*, with big beautiful light blue florets, and a vase life of about 11–12 days (Khunmuang et al., 2016, 2018). The main senescence symptoms were flower wilting, epinasty, petal discoloration, and abscission. Our previous study showed that exposure of three *Vanda* cultivars, ‘Patchara Delight’, ‘Pure Wax’, and ‘Sansai Blue’ to 10 μl L^−1^ exogenous ethylene for 24 h significantly reduced by about 50% their vase life (Khunmuang et al., 2019). Ethylene treatment resulted in partial reduction of the anthocyanin content of ‘Patchara Delight’ after 2 days of vase life, mainly in the full bloom developmental stage, but had no effect on the coloration of ‘Pure Wax’ except in the bud stage. The flowers of ‘Sansai Blue’ showed a fast discoloration that occurred much before the wilting and other senescence symptoms (Khunmuang et al., 2019).

While regulation of anthocyanin biosynthesis at the physiological and molecular levels in flowers has been well studied and documented (Bradley et al., 1998; Weiss, 2000; Quattrocchio et al., 2006; Albert et al., 2009, 2010; Davies et al., 2012; Schwinn et al., 2014), the process of anthocyanin degradation was hardly investigated (Vaknin et al., 2005; Oren-Shamir, 2009). Recent studies reported that vacuolar peroxidases, belonging to the class III peroxidase, were responsible for the *in-planta* degradation of anthocyanins in *Brunfelsia calycina* flowers and in ripening grape berries grown in high temperatures (Zipor et al., 2014; Movahed et al., 2016; Lecourieux et al., 2017; Pastore et al., 2017). Therefore, it was of interest to further investigate in the present study, the rapid color fading in response of cut *Vanda* ‘Sansai Blue’ flowers to exogenous ethylene, focusing on the mechanism of anthocyanin breakdown process.

## Materials and Methods

### Plant Materials and Treatments

Inflorescences of *Vanda* ‘Sansai Blue’ orchid were obtained from a commercial farm in Kanchanaburi province, Thailand. Orchid inflorescences bearing 5–8 open florets and 2–4 buds were selected for the experiment, and transported to King Mongkut’s University of Technology Thonburi (KMUTT), Bangkhuntien campus, Bangkok within 1.5 h. Upon arrival to the laboratory, flower stems were re-cut under water to a 20-cm length from the stem end to the lower first flower.

For application of ethylene, inflorescences in vases with distilled water were exposed either to air as control, or to 1 or 10 μl L^−1^ ethylene for 24 h, in a 43-L glass chamber. The 1-MCP and ethylene treatment was applied by exposing the inflorescences to 0.2 μl L^−1^ 1-MCP (0.14% w:w; Ethylbloc^®^, FloraLife, Walterboro, SC, USA) for 6 h, followed by exposure to 10 μl L^−1^ ethylene for 24 h, in a 43-L glass chamber. All treatments were performed in a controlled environment room, maintained at 21 ± 2°C, 70–80% RH, under cool-white fluorescence light for 12 h day^−1^. After treatments, flowers in vases with distilled water were incubated in the observation room throughout the experimental period.

Individual florets from five different developmental stages (tight bud; colored bud; half bloom; bloom; and full bloom) were detached from the inflorescences immediately or 2 days after treatments, photographed for their visual appearance, and assayed for their anthocyanin content as indicated below.

### Evaluation of Inflorescence Vase Life Longevity

The number of senescing florets in the orchid inflorescences was recorded during the experiment. The vase life was terminated when more than 30% of the florets in an inflorescence lost quality due to petal necrosis, wilting (expressed in loss of turgidity – “sleepiness”), and/or abscission.

### Determination of Anthocyanin Content by Spectrophotometer

Total anthocyanin was extracted and quantified as previously described (Rodriguez-Saona and Wrolstad, 2005). Samples (about 0.1 g) of fresh petals of florets at different developmental stages were grounded under liquid nitrogen to a fine powder, mixed with 10 ml of 0.01% HCl in methanol, and the extracts were incubated overnight in darkness at 4°C. Absorbance of these extracts was monitored at 530 nm using a spectrophotometer (UV-1800 Shimadzu). Anthocyanin content in the samples was expressed as OD530 mg FW^−1^.

### Extraction and Purification of Anthocyanin Samples for HPLC

*Vanda* floret petal samples (approximately 5 g FW) were homogenized in 20 ml of acidified methanol (containing 0.01% HCl), and incubated overnight in darkness at 4°C. The samples were filtered through Whatman no. 1 filter papers. The supernatant was removed, the pellet was re-extracted in 20 ml of acidified methanol, and the extracts were combined. The combined supernatants were dried at 40°C under vacuum (Buchi Evaporator; Vacuum controller V-800: Rotavapor R-205: Heating Bath B-490: Vac^®^V-500: Recirculating chiller B-740). The samples were evaporated until droplet, and the volume of the anthocyanin extract was adjusted to 0.5 ml with acidified water (containing 0.01% HCl).

The anthocyanin extraction-purification method was performed according to Rodriguez-Saona and Wrolstad (2005), with slight modifications. The anthocyanins were purified using a C18 cartridge (Water Sep-Pak^®^). The C18 cartridge was activated by flushing twice with methanol, followed by flushing three times with acidified water. The anthocyanins extract (dissolved in acidified water) was loaded into the activated cartridge, and then washed twice with acidified water to remove sugars, acids, and water soluble compounds, followed by washing with ethyl acetate for elimination of procyanidins. Finally, anthocyanins were eluted by acidified methanol. The purified anthocyanins were concentrated by evaporation at 40°C under vacuum, using a rotary evaporator.

### HPLC Analysis and Composition of Total Anthocyanin

The non-hydrolyzed anthocyanin extracts were analyzed by HPLC (Shimadzu; DGU-20AS Degasser: LC-20AT Liquid Chromatograph: SPD-M20A Photo Diode Array Detector: SIL-20A Auto Sampler: C18 Inertsil^®^ ODS-3; 4.6 mm × 250 mm, 5 μm column). The mobile phase consisted of solvent A (4% phosphoric acid in water) and solvent B (100% acetonitrile). Elution was performed in a linear gradient at the following ratios of solvent A and solvent B – 95:5, 77:23, 77:23, and 95:5 during 1, 25, 29, and 29.01 min, respectively, at a flow rate of 0.7 ml min^−1^ for 40 min. Chromatograms were obtained by LC solution program. Purified anthocyanins were dissolved in 4% phosphoric acid prior to HPLC purification. Anthocyanins were identified by comparing the retention time (RT) and spectral patterns of standard compounds. The content of the main three non-hydrolyzed anthocyanins at RT of 27.6, 33.8, and 36.0 min, were calculated and expressed as cyanidin-3-glucoside equivalents, after running a standard curve in HPLC under the same conditions. The contents were calculated according to the following equation: *Y* = 5586.6*X* − 34,050 (*Y* = peak area; *X* = ng cyanidin-3-glucoside).

For determination of anthocyanidins composition, another sample of purified anthocyanins (1.5–10 mg DW, lyophilized) was hydrolyzed by boiling in 2 N HCl for 1 h, and separated as described by Dela et al. (2003). Hydrolyzed anthocyanin samples were analyzed by HPLC (Shimadzu, Japan) equipped with an LC-10AT, an SCL-10A, and an SPD-M10AVP photodiode array detector. Separation was performed on a RP-C18 column (201TP54, Grace Vydac) at 27°C with the following solutions: (A) H_2_O, pH 2.3 and (B) H_2_O:MeCN:HOAc (107:50:40), pH 2.3. The solutions were applied as a linear gradient from a ratio of 4:1 (A:B) to 3:7 over 45 min, and held at a ratio of 3:7 for an additional 10 min at a flow rate of 0.5 ml min^−1^. Anthocyanidins and flavonols were identified by comparing both the RT and the absorption spectrum from 250 to 650 nm to those of standard purified anthocyanidins and flavonols (obtained from Apin Chemicals, UK; Polyphenols, Norway; Sigma Aldrich, USA).

### Extraction and Determination of Peroxidase Activity

Peroxidase (POD) activity of *Vanda* florets was determined as previously described (Gerailoo and Ghasemnezhad, 2011), with some modifications. Enzyme extraction for POD activity was prepared by homogenization of 2 g of floret petal samples in 20 ml extraction buffer composed of 50 mM phosphate buffer, pH 7, and 1% PVPP (w/v). The samples were centrifuged for 30 min at 12,000× *g*, and the supernatant was used to determine enzyme activity. POD activity was assayed by measuring spectrophotometrically the formation of guaiacol in l ml reaction mixture composed of 450 μl guaiacol 25 mM, 450 μl H_2_O_2_ 225 mM, and 1 ml crude enzyme. The formation of tetraguaiacol was measured at 470 nm, and the activity was expressed as units per mg protein. Protein concentrations were measured as described by Bradford (1976).

### Statistical Analysis

Experiments were arranged in a completely randomized design (CRD), with 6–8 replicate stems for each treatment. Data were analyzed using ANOVA, and differences among means were compared using Tukey Test.

## Results

**Figure 1:**
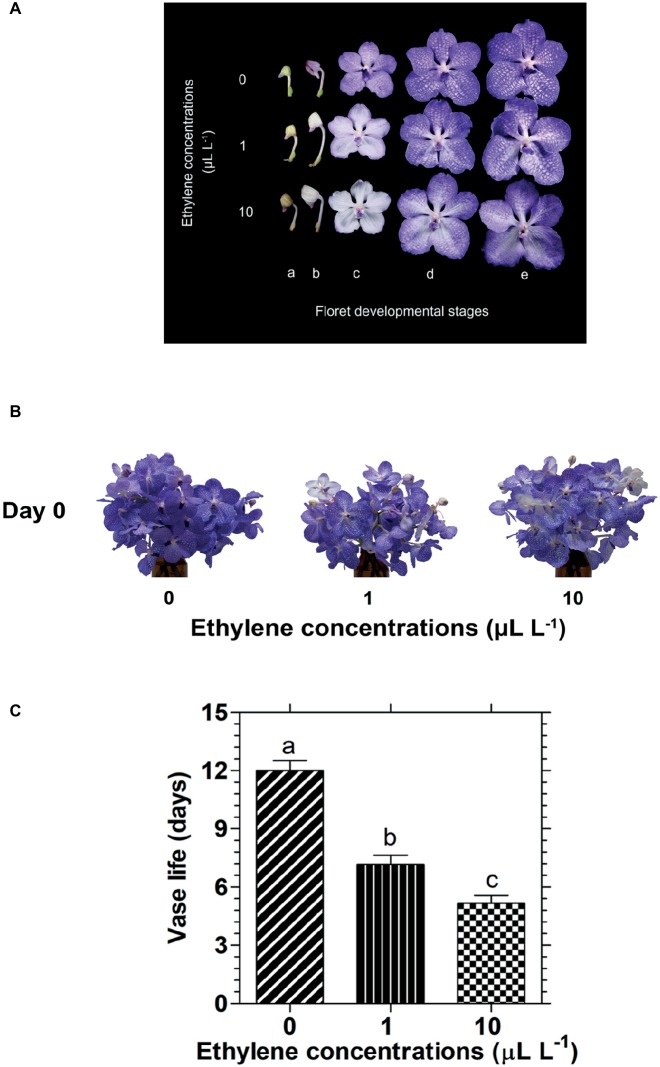
Effect of exposure of cut *Vanda* ‘Sansai Blue’ flowers to different ethylene concentrations on the visual appearance of florets at various developmental stages **(A)**, on the visual appearance of cut inflorescences photographed immediately after removal from the ethylene environment (day 0) **(B)**, and on their vase life longevity **(C)**. The inflorescences were exposed to 0 (control), 1, or 10 μl L^−1^ ethylene for 24 h at 21 ± 2°C, and then placed in the observation room. The different floret developmental stages were defined as follows: a, tight bud; b, colored bud; c, half bloom; d, bloom; e, full bloom. The results in graph **(C)** represent means ± SE of 6–8 inflorescence replicates per treatment. Different letters indicate significant differences at *p* < 0.01.

Exposure of cut *Vanda* ‘Sansai Blue’ inflorescences to two ethylene concentrations for 24 h resulted in a dramatic bleaching of the florets at development stages B (colored bud) and C (half bloom) already during the 24 h of ethylene treatment (Figure 1A). On the other hand, in the more developed stages, D (bloom) and E (full bloom), the decrease in petal pigmentation was less noticeable at the end of the ethylene exposure (Figure 1A). Indeed, the appearance of a bunch of inflorescences held in the vase after their removal from the atmosphere of both ethylene concentrations, showed a faded color compared to control, but they were still blueish because most florets were in stages D and E (Figure 1B). It should be noted that the fast bleaching of the florets at developmental stages B and C (Figure 1A), and the visual color appearance of the whole inflorescences at day 0 (Figure 1B) were similar for the two ethylene concentrations. By contrast, the significant reduction in their vase life longevity following exposure to ethylene was concentration-dependent (Figure 1C). Thus, exposure of the inflorescences to 1 or 10 μl L^−1^ ethylene significantly shortened their vase life to 7.2 and 5.2 days, respectively, as compared to control flowers which lasted for 12 days of vase life (Figure 1C).

**Figure 2:**
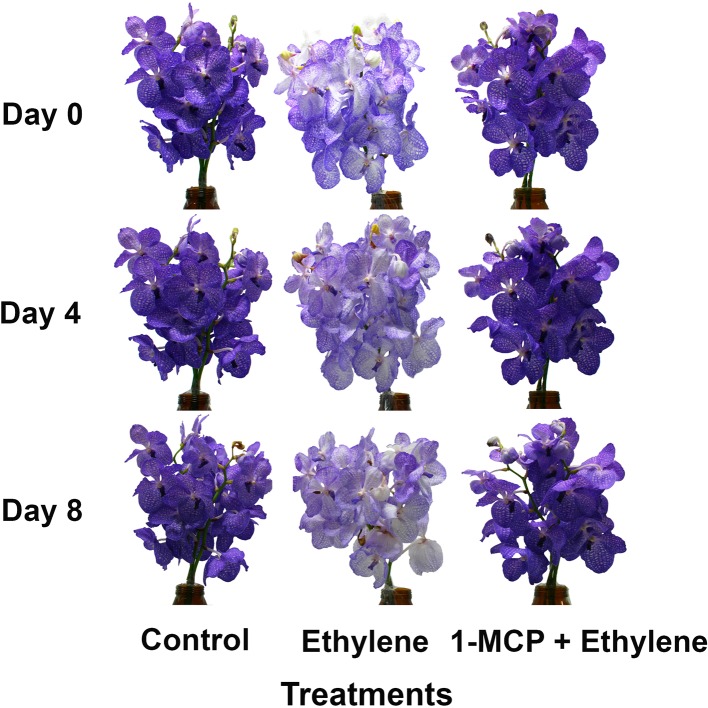
Effect of ethylene and 1-MCP pre-treatments on the visual appearance of inflorescences during vase life of cut *Vanda* ‘Sansai Blue’ flowers after treatment application. The inflorescences were exposed to air (control), 10 μl L^−1^ ethylene for 24 h, or to 0.2 μl L^−1^ 1-MCP for 6 h followed by 10 μl L^−1^ ethylene for 24 h at 21 ± 2°C, and then placed in the observation room.

In order to further examine the effect of ethylene on flower pigmentation during vase life, the inflorescences were exposed to 10 μl L^−1^ ethylene or to 0.2 μl L^−1^ 1-MCP followed by 10 μl L^−1^ ethylene. The results depicted in Figure 2 demonstrate that the color intensity of the ethylene-treated flowers dramatically decreased on days 4 and 8, and this effect was completely inhibited by the 1-MCP pretreatment, which recovered the color appearance to that of control flowers at these time points (Figure 2).

**Figure 3:**
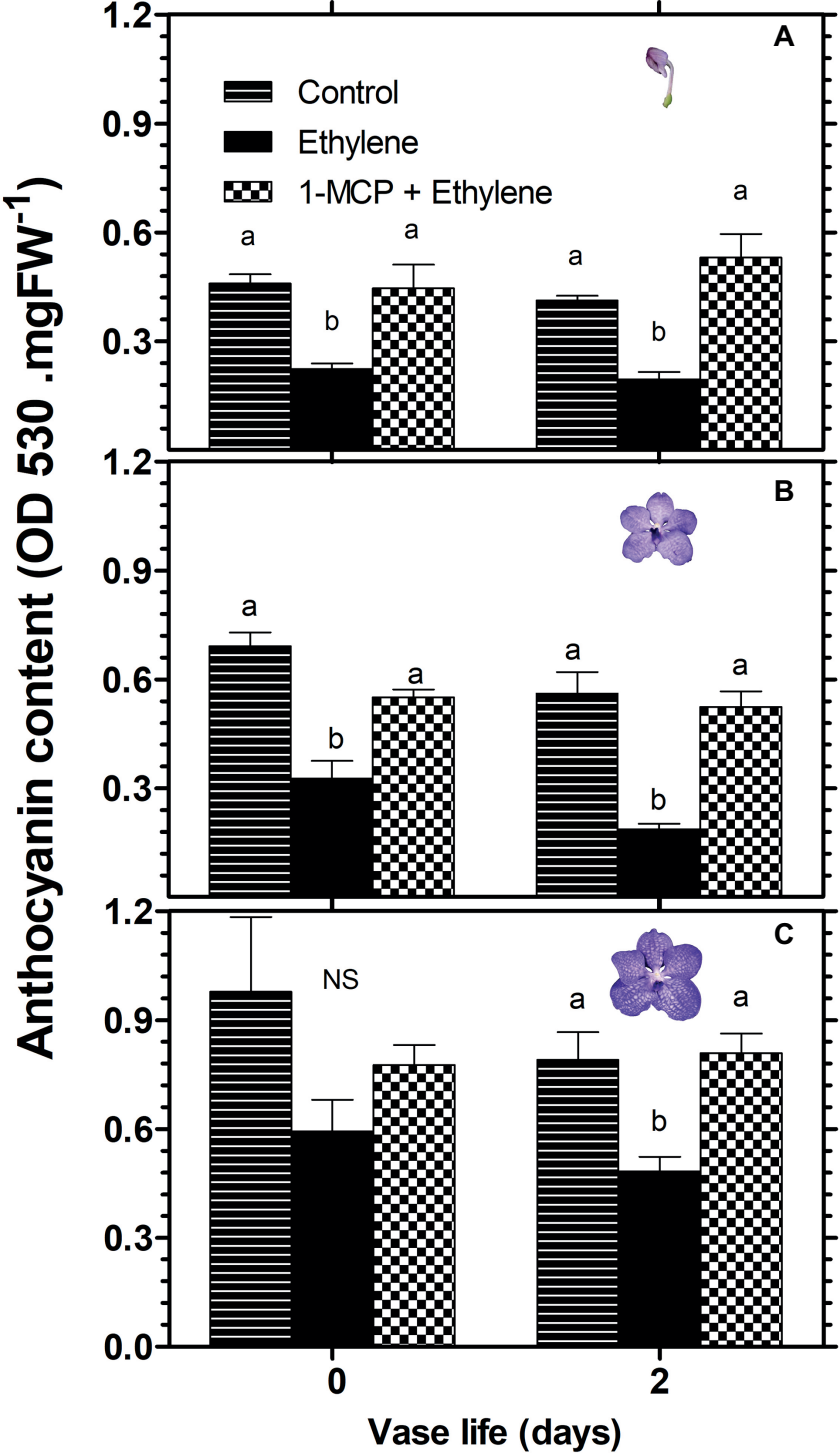
Effect of ethylene and 1-MCP pre-treatments on changes in anthocyanin content of cut *Vanda* ‘Sansai Blue’ flowers at different developmental stages of colored bud **(A)**, half bloom **(B)**, and bloom **(C)**, during 2 days of vase life after treatments. Ethylene and 1-MCP pre-treatments were applied as detailed in Figure 2, and florets at the indicated developmental stages were sampled and assayed spectrophotometrically for anthocyanin content. The results represent means ± SE of four floret replicates per treatment. Different letters indicate significant differences among treatments at the different time points, at *p* < 0.05 for day 0, or at *p* < 0.01 for day 2 (graph **A**); at *p* < 0.01 (graphs **B,C**); NS, not significant.

A similar pattern of changes in response to these treatments was obtained in the anthocyanin content of florets, analyzed during 2 days of vase life after treatment. Thus, ethylene treatment significantly reduced the anthocyanin content of florets at the developmental stages of colored bud (Figure 3A) and half bloom (Figure 3B) on day 0, while the ethylene-induced reduction was less significant for florets at bloom stage (Figure 3C). On the other hand, on day 2, the reduction in anthocyanin content was significant for all three developmental stages (Figure 3). 1-MCP pretreatment abolished completely the ethylene effect on the anthocyanin content in all floret development stages at both time points, and recovered the anthocyanin levels to those of control florets (Figure 3).

**Figure 4:**
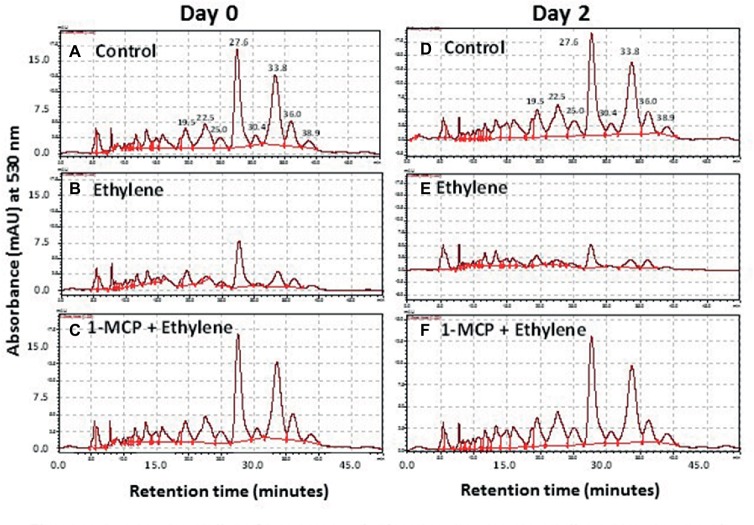
Representative chromatograms of non-hydrolyzed anthocyanins, extracted from *Vanda* cv. ‘Sansai Blue’ florets, showing changes in the content of the two main anthocyanin peaks in response to ethylene and 1-MCP pre-treatments, immediately **(A–C)** and 2 days **(D–F)** after treatments. Anthocyanins were extracted from control **(A,D)**, ethylene-treated **(B,E)** or 1-MCP, and ethylene-treated **(C,F)** florets at the bloom developmental stage (see Figure 1A). Ethylene and 1-MCP pre-treatments were applied as detailed in Figure 2.

More than eight anthocyanin peaks were observed in the chromatogram of *Vanda* ‘Sansai Blue’ florets at bloom stage following the HPLC analysis of non-hydrolyzed samples (Figure 4A). Two major anthocyanins were separated at RT of 27.6 and 33.8 min, and six less abundant anthocyanins appeared at RT of 19.5, 22.5, 25.0, 30.4, 36.0, and 38.9 min. The content of all these anthocyanins (peak height and area) remained similar in control florets at bloom stage after 2 days of vase life (Figure 4D). A drastic decrease of all anthocyanins could be observed in the ethylene-treated flowers, when flowers were removed from the ethylene atmosphere (day 0) (Figure 4B), and the small remaining residues of anthocyanin peaks further decreased to almost nullified levels on day 2 (Figure 4E). 1-MCP pretreatment prevented completely and very efficiently the ethylene-enhanced effect on the anthocyanin degradation both on day 0 (Figure 4C), and on day 2 (Figure 4F). Thus, the 1-MCP pretreatment recovered the anthocyanin levels to those of control untreated flowers (Figures 4A,D).

**Figure 5:**
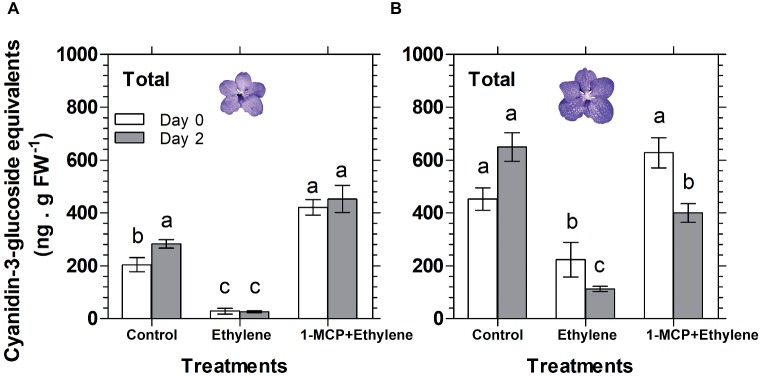
Effect of ethylene and 1-MCP pre-treatments on changes, detected 2 days after treatments, in the content of total anthocyanins extracted from florets at half bloom **(A)** or bloom **(B)** developmental stages, based on the chromatogram peak areas of non-hydrolyzed samples of *Vanda* ‘Sansai Blue’ florets. Ethylene and 1-MCP pre-treatments were applied as detailed in Figure 2, and the anthocyanin level was calculated as cyanidin-3-glucoside equivalents. The results represent means ± SE of three floret replicates per treatment, and different letters indicate significant differences among treatments at the different time points, at *p* < 0.01.

Similar results were obtained also for florets analyzed at the half bloom stage, in which the anthocyanins were nullified already on day 0 (data not shown). A quantitative data for the HPLC analysis described above is presented in Figure 5 for total anthocyanins (the sum of peak area of all anthocyanins), expressed as cyanidin-3-glucoside equivalents. The data show that the ethylene treatment nullified the content of total anthocyanins during the ethylene exposure of florets at half bloom stage (Figure 5A), while at the bloom stage there was a continuous degradation of anthocyanins in response to ethylene between day 0 and day 2 (Figure 5B). 1-MCP pretreatment prevented the ethylene-induced anthocyanin degradation, and the levels of anthocyanins at half bloom stage even increased over control levels (Figure 5A), suggesting that anthocyanins were also synthesized during this period.

**Figure 6:**
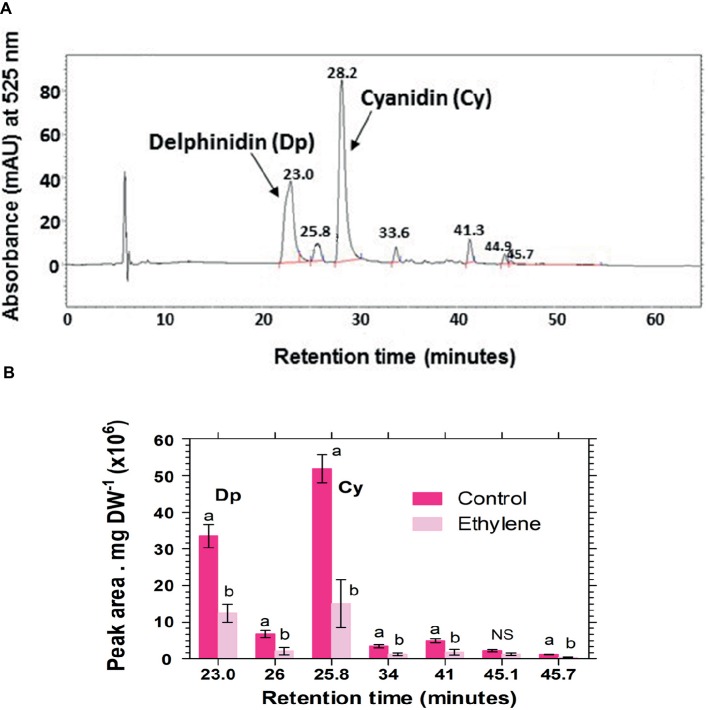
A typical chromatogram profile of anthocyanidins detected in control hydrolyzed samples of *Vanda* ‘Sansai Blue’ florets **(A)**, and effect of ethylene treatment on the levels of these anthocyanidins, detected 2 days after treatment **(B)**. The inflorescences were exposed either to air (control) or to 10 μl L^−1^ ethylene for 24 h at 21 ± 2°C. Anthocyanins were extracted from florets at the bloom developmental stage (see Figure 1A), 2 days after treatment, hydrolyzed, purified and chromatographed. The results in graph **(B)** represent means ± SE of three floret replicates per treatment, and different letters indicate significant differences among treatments at the different time points, at *p* < 0.01 (for 21.44, 25.57, 45.7 min RT), or at *p* < 0.05 (for 37.43, 28.4, 34.37 min RT); NS, not significant. Dp, Delphinidin; Cy, Cyanidin.

**Figure 7:**
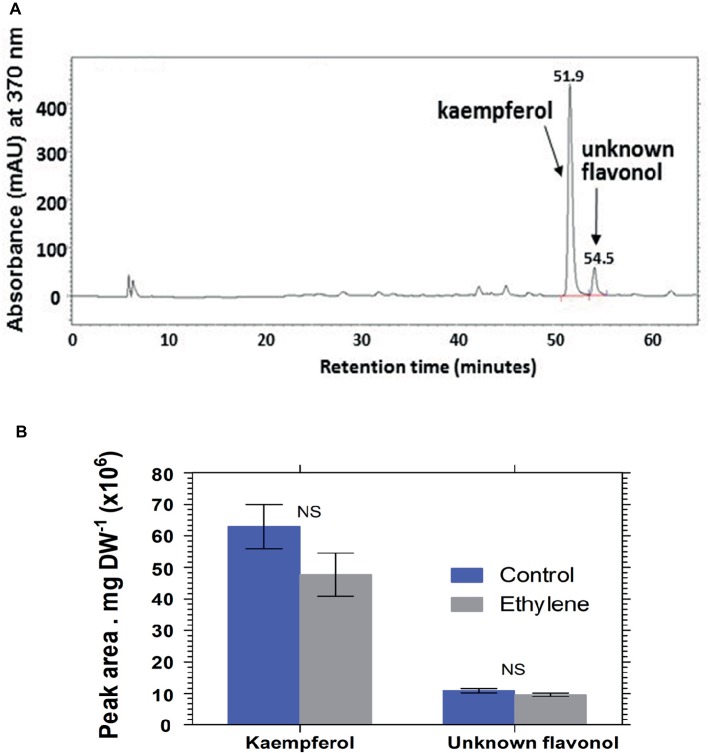
A typical chromatogram profile of flavonols detected in control hydrolyzed samples of *Vanda* ‘Sansai Blue’ florets **(A)**, and effect of ethylene treatment on the levels of these flavonols detected 2 days after treatment **(B)**. The inflorescences were exposed either to air (control) or to 10 μl L^−1^ ethylene for 24 h at 21 ± 2°C. Flavonols were extracted from florets at the bloom developmental stage (see Figure 1A), 2 days after treatment, hydrolyzed, purified, and chromatographed. The results in graph **(B)** represent means ± SE of three floret replicates per treatment. NS, not significant.

Analysis of hydrolyzed anthocyanin samples revealed that most of the anthocyanin pigments in *Vanda* ‘Sansai Blue’ flowers were based on delphinidin and cyanidin backbones (Figure 6). The results confirmed the previous findings, and show that ethylene reduced all anthocyanidins during the treatment at bloom stage florets (Figure 6B). Kaempferol was found to be the dominant flavonol in *Vanda* ‘Sansai Blue’ flowers (Figure 7), but unlike the anthocyanidins, the flavonols were not affected by the ethylene treatment (Figure 7B).

**Figure 8:**
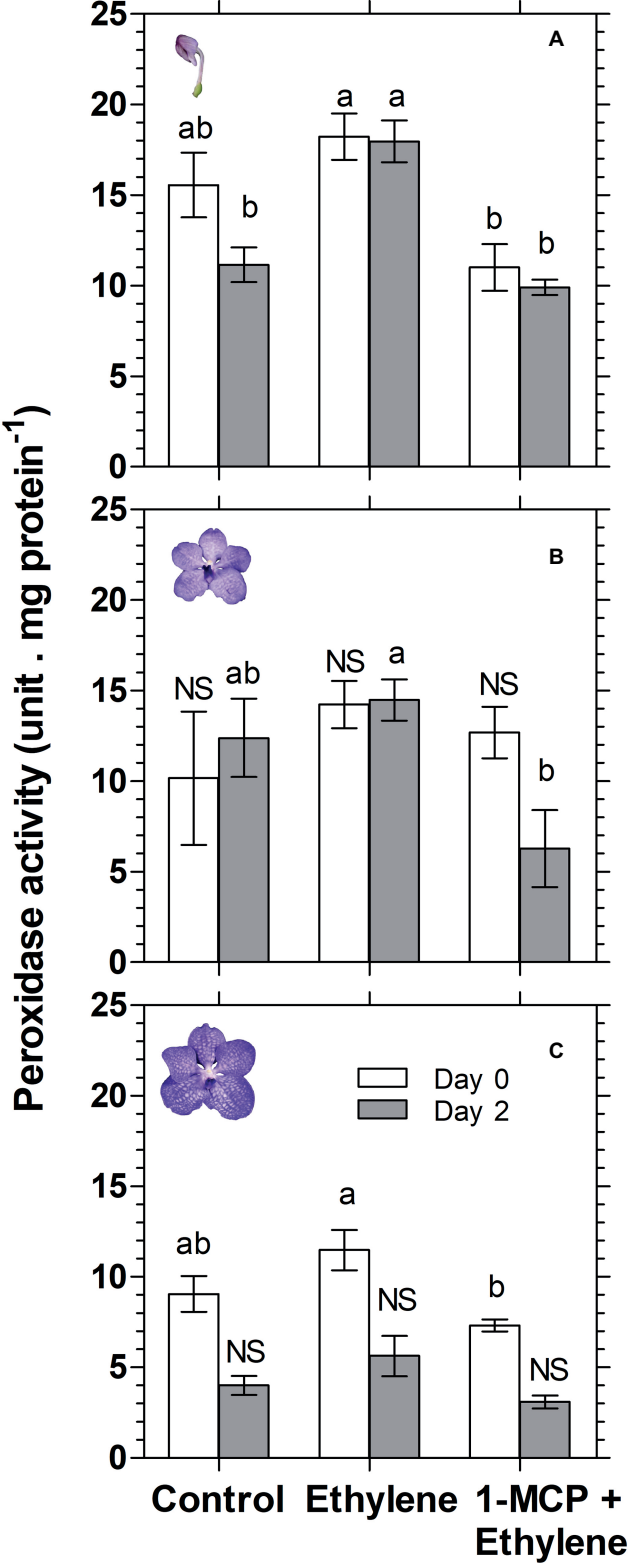
Effect of ethylene and 1-MCP pre-treatments on changes in total peroxidase (POD) activity of *Vanda* ‘Sansai Blue’ cut flowers assayed at different developmental stages of colored bud **(A)**, half bloom **(B)**, and bloom **(C)**, during 2 days of vase life after treatments. Ethylene and 1-MCP pre-treatments were applied as detailed in Figure 2, and florets at the indicated developmental stages were sampled and assayed for POD activity. The results represent means ± SE of four floret replicates per treatment. Different letters indicate significant differences among treatments at the different time points, at *p* < 0.05 for day 0, or at *p* < 0.01 for day 2 (graph **A**); at *p* < 0.01 (graph **B**); at *p* < 0.05 for day 0 (graph **C**); NS, not significant.

Anthocyanin degradation was reported to be mediated by enzymatic activity of the class III peroxidase (POX) (Zipor et al., 2014; Movahed et al., 2016; Lecourieux et al., 2017; Pastore et al., 2017). Therefore, in an attempt to investigate the mechanism of anthocyanin degradation in cut *Vanda* ‘Sansai Blue’ flowers, we have examined the effect of ethylene treatment on total peroxidase (POD) activity at different developmental stages (Figure 8). The results show a significant ethylene-induced increase in POD activity on day 2 in the colored bud stage (Figure 8A). 1-MCP pretreatment resulted in the lower POD activity, generally at all developmental stages, and it inhibited completely the ethylene-induced increase in POD activity (Figure 8). These results suggest that the ethylene-induced anthocyanin degradation in the cut *Vanda* ‘Sansai Blue’ flowers seems to be mediated by increased POD activity.

## Discussion

### Ethylene Induces Color Fading in *Vanda* Flowers Independently of Senescence

The effect of exogenous ethylene treatment on cut *Vanda* ‘Sansai Blue’ flowers was very dramatic and rapid, and was already pronounced during the 24 h of exposure to the ethylene atmosphere (Figures 1A,B). In the younger flower developmental stages of colored buds and half bloom, the florets became almost completely white, while in the more advanced developmental stages, the bleaching was only partial during the ethylene treatment (Figure 1B), and the pigment degradation continued to take place throughout vase life (Figure 2). Both ethylene concentrations of 1 and 10 μl L^−1^ induced the same degree of color fading (Figures 1A,B, 2), indicating that the fading-induced effect of ethylene was not concentration-dependent in this range. On the other hand, the effect of ethylene treatment on flower senescence, expressed in a significant reduction of vase life due to floret wilting, was dependent on ethylene concentrations (Figure 1C). This suggests that the two processes of the rapid ethylene-induced anthocyanin degradation and the ethylene-induced senescence, proceed as separate processes at a different timing in cut *Vanda* ‘Sansai Blue’ flowers.

This conclusion is further supported by the following additional observations: (1) the wilting of the florets occurred first in the florets at the more advanced developmental stages, which are located at the bottom of the inflorescence, and proceeded upward. However, the upper florets that became completely bleached were still turgid and continued to bloom and grow until the end of the experiment (Figure 2). The decrease in water uptake in the ethylene-treated flowers was the reason for the enhanced decrease of the inflorescences FW, but the first wilted florets at the bottom of the inflorescence could be observed only when their FW decreased to about 93% of the initial value (Khunmuang et al., 2016, 2018). (2) The ethylene-induced bleaching of cut *Vanda* ‘Sansai Blue’ flowers was not accompanied by wilting symptoms or any other well-documented senescence parameters, such as ion leakage, protein degradation, and increased amino acid content (Mayak, 1987; Van Doorn, 2001; Van Doorn and Woltering, 2008; Rogers, 2013; Dar et al., 2014), during more than 7 days after treatment (Khunmuang et al., 2019). The presented results further suggest that the rapid ethylene-induced anthocyanin degradation of cut *Vanda* ‘Sansai Blue’ flowers proceeds as a separate process, independently from the well-characterized senescence-associated processes.

A similar fast ethylene-induced color fading was reported long ago in *Vanda* flowers, but in an indirect manner in response to pollination or emasculation, which induced high levels of endogenous ethylene production (Akamine, 1963; Burg and Dijkman, 1967; Goh et al., 1985). Thus, emasculation of *Vanda* ‘Rose Marie’ resulted in increased ethylene evolution, which started after a 10-h lag period, and fading became evident after additional 8–12 h (Burg and Dijkman, 1967). A similar time course of ethylene evolution and fading was reported for *Vanda* ‘Miss Agnes Joaquim’ flowers after their emasculation, in which ethylene production was correlated with the degree of color fading (Akamine, 1963). In *Vanda* ‘Petamboerant’ flowers, endogenous ethylene production rates increased in control flowers after 75 h, while pollination or emasculation enhanced the process by inducing ethylene production within 1 or 28 h, respectively (Burg and Dijkman, 1967). Consequently, the lower petals of pollinated or emasculated *Vanda* ‘Petamboeran’ flowers started to fade after 8–10 or 35 h, respectively, as compared to petals of control flowers which faded after about 80 h (Burg and Dijkman, 1967). Also in other orchid flowers of *Phalaenopsis* (Porat et al., 1995) and *Dendrobium* ‘Pompadour’ (Ketsa and Rugkong, 2000), pollination induced endogenous ethylene production, which enhanced their senescence.

A more direct effect of exogenous ethylene was reported recently, describing pulsing of cut *Vanda* ‘Sansai Blue’ flowers with the ethylene-releasing compound, ethephon. This treatment resulted in reduced water uptake and vase life longevity, and this effect was prevented by 1-MCP pretreatment, but the effect on color bleaching was less visible (Khunmuang et al., 2016). This discrepancy between the treatments could be ascribed to the fact that the ethylene released by ethephon pulsing affected more the stems, and less directly the florets at the younger developmental stages. In other orchid flowers, application of 10 μl L^−1^ ethephon by dipping for 5 min decreased significantly the water uptake of cut *Dendrobium* ‘Planty Fushia’ flowers, and reduced their vase life. Termination of vase life was due to senescence and abscission of the open florets at the bottom of the inflorescences, and prevention of opening of the florets at the bud stages that were abscised (Mohammadpour et al., 2015). These ethephon effects on cut *Dendrobium* “Planty Fushia” flowers were completely inhibited by 1-MCP pretreatment. Application of 0.25–2 μl L^−1^ 1-MCP to cut *Dendrobium* ‘Burana Jade’ flowers was even more effective in maintaining the FW as compared to control flowers (Yoodee and Obsuwan, 2013). This indicates that 1-MCP is effective also in inhibition of endogenous ethylene.

### Anthocyanin Degradation Is Responsible for the Rapid Color Fading

The visible bleaching of the cut *Vanda* ‘Sansai Blue’ flowers (Figures 1A,B, 2) was due to anthocyanin pigment degradation induced by the ethylene treatments (Figure 3). The reduction in anthocyanin content in florets at half bloom and bloom stages in response to ethylene was quantitatively quite similar during the ethylene treatment (Figure 3). The light blueish color (Figure 1B) on day 0 and during vase life (Figure 2) observed in the ethylene-treated flowers reflected the pigment residues in the florets at more advanced developmental stages. The initial content of the anthocyanins in the more advanced developmental stages (Figure 3C) was much higher than in the early developmental stages (Figure 3A), and the anthocyanins continued to decrease in the ethylene-treated flowers (Figures 2, 3). The higher anthocyanin contents in the more developed floret stages (Figure 3C) indicate that anthocyanins continued to be synthesized in the flowers until reaching the full bloom stage. 1-MCP pretreatment completely inhibited the ethylene-induced floret bleaching (Figure 2), and anthocyanin degradation (Figures 3–5). Indeed, the visual appearance on day 8 of inflorescences pretreated with 1-MCP shows that their color was more pronounced than that of the control untreated flowers (Figure 2), but on the other hand, more florets with “sleepiness” symptoms at the bottom were detected (Khunmuang et al., 2019).

The results presented in Figure 3 clearly demonstrate that anthocyanin content increases with development (zero time in each stage), but this process takes time (about 10 days from bud to a fully open flower). On the other hand, during the initial 2 days of vase life, anthocyanin levels did not change in the control samples of each developmental stage, but they were reduced significantly in the ethylene-treated samples, and this reduction was inhibited by 1-MCP. These results clearly show that the decreased anthocyanin levels resulted from the ethylene-induced degradation rather than from inhibition of anthocyanin biosynthesis.

Our previous study showed that while the effect of ethylene treatment in reducing the vase life longevity was similar in three cut *Vanda* cultivars, the effect of ethylene on flower color bleaching and anthocyanin content varied among the cultivars and floret stages (Khunmuang et al., 2019). The anthocyanin content of *Vanda* ‘Pure Wax’ flowers was almost unaffected by ethylene, except at the bud stage, while in cut *Vanda* ‘Patchara Delight’ flowers it was partially reduced after 2 days of vase life, mainly in the full bloom developmental stage. This suggests that the three *Vanda* cultivars differ in their sensitivity to ethylene, which is expressed at different developmental stages, and the *Vanda* ‘Sansai Blue’ flowers are unique among the three cultivars in their high sensitivity to ethylene manifested in the rapid color fading.

Variation in ethylene sensitivity may be related to differences in the concentration and affinity of the ethylene receptors and/or to the activity of downstream components in the signal transduction pathway, which activates gene transcription and translation (Bleecker and Kende, 2000). It was previously suggested that *Vanda* ‘Sansai Blue’ flowers are sensitive to ethylene but the flower itself produced very low amount of ethylene (Goh et al., 1985; Khunmuang et al., 2016, 2018).

### Ethylene Induces Degradation of Anthocyanins *in planta*

More than eight anthocyanins were observed in *Vanda* ‘Sansai Blue’ flowers analyzed at the bloom stage by HPLC of unhydrolyzed samples, and all of them were fast degraded by ethylene treatment (Figure 4). Pigment accumulation, anthocyanin structure and the expression of floral anthocyanin genes were analyzed in anthocyanin-based colored florets of a pale-mauve *Vanda* hybrid (*V. teres* × *V. hookeriana*) (Junka et al., 2011). The anthocyanins gradually accumulated during all the developmental stages of the florets. Based on HPLC and LC-ESI-MSn analyses, the anthocyanins in the pale-mauve hybrid were composed of only five types of cyanidin derivatives, which were diversely conjugated with some hexose sugars and organic acids, such as ferrulic, sinapic, and malonic acids (Junka et al., 2011). On the other hand, more than 11 anthocyanins were observed in the violet-blue and red-purple flowers of the *Vanda* hybrid cultivars, from which eight major acylated anthocyanins were isolated (Tatsuzawa et al., 2004). Four of those pigments were based on cyanidin 3,7,3′-triglucoside, and the other four pigments were based on delphinidin 3,7,3′-triglucoside as their deacylanthocyanins. The distribution of these pigments was investigated in the flowers of four species and 13 hybrids by the analytical process of HPLC. Unfortunately, the *Vanda* ‘Sansai Blue’ was not included in this survey, but our anthocyanins hydrolyzed extract analysis revealed that most anthocyanidins in this cultivar are based on both delphinidin and cyanidin as well (Figure 6). The acylated anthocyanins of cyanidin and delphinidin contribute to make the blue flower color in the *Vanda* cultivars, as well as the presence of delphinidin glycosides (Tatsuzawa et al., 2004). This is generally true according to previous studies of orchids, which indicated that the bluing effect was dependent on the numbers of hydroxycinnamic acids (Lu et al., 1992; Honda and Saito, 2002).

The main flavonols that were found in *Vanda* ‘Sansai Blue’ florets were kaempherol and another unknown flavonol, which were not affected by the ethylene treatment (Figure 7). It seems, therefore, that they are not involved in the ethylene-induced color fading. Based on these results, it is clear that the ethylene-induced bleaching of *Vanda* ‘Sansai Blue’ flowers occurring *in planta* is ascribed only to the degradation of anthocyanins and not of flavonols.

### Ethylene-Induced Anthocyanin Degradation Is Mediated by Peroxidase

Anthocyanin degradation has been detected *in vivo* in some systems such as the loss of red pigmentation in maturing leaves of *Photinia* spp. (Oren-Shamir and Nissim-Levi, 1999). The enzymatic degradation hypothesis was strongly supported by the investigation on *B. calycina* Benth., in which active anthocyanin degradation by oxidation was reported *in planta* (Vaknin et al., 2005; Oren-Shamir, 2009; Zipor et al., 2014). This loss of color from dark purple to white was dependent on anthocyanin degradation, and *de novo* synthesis of mRNAs and proteins during the different stages of development, well before flower senescence has started (Vaknin et al., 2005), similar to the results reported in the present study for *Vanda* ‘Sansai Blue’ flowers. A candidate peroxidase was partially purified and characterized, its intracellular localization was determined for *B. calycina* flowers (Zipor et al., 2014), and the transcript sequence of this peroxidase was fully identified. A basic peroxidase, BcPrx01, was responsible for the *in planta* degradation of anthocyanins in *B. calycina* flowers. BcPrx01 had the ability to degrade complex anthocyanins, it co-localized with these pigments in the vacuoles of petals, and both the mRNA and protein levels of BcPrx01 were greatly induced in parallel to the degradation of anthocyanins (Zipor et al., 2014). Recent studies confirmed the degradation of anthocyanins *in planta* by peroxidases, which exhibited higher activity at elevated temperatures (Movahed et al., 2016; Lecourieux et al., 2017; Pastore et al., 2017). Overexpression of the grapevine peroxidase gene (*Vvi-Prx31*) decreased anthocyanin contents in *Petunia hybrida* petals under heat stress condition, suggesting that a high temperature can stimulate peroxidase activity and anthocyanin degradation in ripening grape berries (Movahed et al., 2016). BcPrx01 and Vvi-Prx3 are vacuolar peroxidases, belonging to the class III peroxidase family (POX), and are able to catalyze the reduction of toxic H_2_O_2_ that reaches the vacuoles by oxidizing a variety of secondary metabolites (Hiraga et al., 2001).

Class III plant peroxidases (POXs) are plant-specific oxidoreductase, which participate in lignification, suberization, auxin catabolism, wound healing, and defense against pathogen infection (Hiraga et al., 2001). Studies have provided information on the regulatory mechanisms of wound- and pathogen-induced expression of some *POX* genes. These studies suggest that *POX* genes are induced *via* different signal transduction pathways from those of other known defense-related genes (Ishige et al., 1993; Ito et al., 1994). Furthermore, high temperature (35°C) increased ethylene production and concentration of H_2_O_2_, and the activity of POX in plum fruit (Niu et al., 2017). It seems, therefore, that ethylene is an enhancer of peroxidase activity. It is important to emphasize that in the present study, total peroxidase (POD) was assayed, rather than the specific class III peroxidase (POX). This may explain the insignificant effects of ethylene on peroxidase activity obtained on day 0 at all assayed developmental stages (Figure 8). Nevertheless, we could demonstrate that ethylene treatment significantly increased total POD activity on day 2 at the bud developmental stage, and this effect was inhibited completely by 1-MCP pretreatment (Figure 8A). These results are consistent with the results of anthocyanin content at the bud stage (Figure 3A). This may indicate that POD activity is highly affected by ethylene at the bud stage, relative to the other developmental stages. Since the anthocyanins accumulate in the vacuole, it is necessary that the vacuolar peroxidase (POX) will degrade them. Our results indicate that at the bud stage, the activity of POX in the POD extract is relatively high, and therefore the results for the ethylene effects at this stage were significant (Figure 8A). Alternatively, since the anthocyanins are synthesized in the cytoplasm at the bud stage, it is still possible that they are degraded by the cytoplasmic peroxidase in response to ethylene, before reaching the vacuole. Additionally, the insignificant effects of ethylene on POD activity obtained at the other developmental stages (Figures 8B,C), may indicate that another unknown mechanism is possibly involved in this process, which needs a further study.

Taken together, our results suggest that the ethylene-induced anthocyanins degradation in cut *Vanda* ‘Sansai Blue’ flowers seems to be mediated by increased POD activity *in-planta*. This effect, which is fast and independent of the flower senescence process, is mainly expressed at the bud developmental stage, in which the anthocyanin degradation was most prominent.

## Data Availability

All datasets generated for this study are included in the manuscript and/or the supplementary files.

## Author Contributions

SKh, MB, SM, SP-H, CW-A, and SKa were responsible for the conception, design of the experiments, and interpretation of data. SKh performed the laboratory experiments and the HPLC analyses. MO-S and RO were responsible for the anthocyanin analyses and determination. SKh, MB, SM, and SP-H were involved in drafting the work. SP-H and SM were responsible for the writing, editing, and final approval of the version to be published. All authors revised and approved the final version.

### Conflict of Interest Statement

The authors declare that the research was conducted in the absence of any commercial or financial relationships that could be construed as a potential conflict of interest.
